# Poly-L-lysine as an Effective and Safe Desquamation Inducer of Urinary Bladder Epithelium

**DOI:** 10.3390/polym11091506

**Published:** 2019-09-16

**Authors:** Mojca Kerec Kos, Peter Veranič, Andreja Erman

**Affiliations:** 1 University of Ljubljana, Faculty of Pharmacy, Askerceva cesta 7, 1000 Ljubljana, Slovenia; kerecm@ffa.uni-lj.si; 2 University of Ljubljana, Faculty of Medicine, Institute of Cell Biology, Vrazov trg 2, 1000 Ljubljana, Slovenia; peter.veranic@mf.uni-lj.si

**Keywords:** poly-l-lysine, urothelium, transepithelial electrical resistance, cell desquamation, ex vivo, in vivo

## Abstract

Induced desquamation of urinary bladder epithelial cells, also called urothelial cells, is frequently used in studies of bladder epithelial regeneration and also in treating recurrent bacterial cystitis. Positively charged polymer chitosan is known to cause large-scale desquamation of terminally differentiated urothelial cells called umbrella cells. Aiming to compare the desquamation ability of another polycation poly-L-lysine, we studied the effect of this polymer on the functional and structural integrity of the urothelium in ex vivo and in vivo experiments. The urothelium was analyzed by measuring transepithelial electrical resistance, and the structural changes of its luminal surface were analyzed with scanning electron microscopy. The results revealed a selective and concentration-dependent desquamation effect of poly-L-lysine on superficial urothelial cells followed by quick regeneration of the urothelium, which functionally and structurally recovers in 2 to 3 h after poly-L-lysine–induced injury. Poly-L-lysine was thus proven to be a promising polymer to be used when desquamation of urothelial cells is required in basic and potentially clinical studies.

## 1. Introduction

The urinary bladder epithelium, called the urothelium, is considered to be one of the most impermeable epithelia in the body and it is a very effective blood–urine barrier [1,2]. The urothelium consists of three cell layers: superficial, intermediate, and basal cells. Under physiological conditions, the urothelium has a very low turnover rate. However, after an injury, undifferentiated basal and partially differentiated intermediate cells rapidly proliferate and differentiate into terminally differentiated superficial cells, also called umbrella cells [3,4,5].

Due to its mucoadhesion, permeability enhancement, and antimicrobial activity, chitosan is widely used in medical research [6,7]. Many drug delivery systems with chitosan intended for intravesical application have been developed, aiming to increase the residence time of the drug in the bladder, ensure better delivery of the drug to the target cells, and avoid systemic side effects [8,9,10,11]. Our previous studies showed that chitosan induces desquamation of the urothelial cells, followed by rapid regeneration of the urothelium in ex vivo and in vivo conditions [4,5]. The influence of chitosan on the urothelial structure and function is dependent on time and concentration, with more pronounced damage at longer exposure and higher concentrations of the polymer [12]. Desquamation of the urothelium induced by chitosan could be beneficial in the case of recurrent urinary tract infections. During urinary tract infection, uropathogenic *Escherichia coli* can persist within the urothelial cells, and these latent intracellular reservoirs are not affected by antibiotic treatments [13]. The repeated use of chitosan together with quinolone antibiotic ciprofloxacin eradicated uropathogenic *Escherichia coli,* which could prevent relapsing bacteriuria [14].

The polymer charge plays an important role in the ability of the polymer to interact with the urothelial surface. In ex vivo experiments, positively charged polymers such as poly-L-arginine and chitosan had the most pronounced influence on the permeability of the urothelium [15]. In a study performed on the intestinal Caco-2 cell line, all polycations investigated (chitosan, polyethylenimine, and poly-L-lysine) induced an increase in tight junction permeability associated with changes in the F-actin cytoskeleton and in the localization of tight junctional proteins [16].

In spite of the fact that chitosan proved to be very effective as a co-treatment with antibiotics in fighting bacterial cystitis [14], the low pH value around 4.5 required to disperse chitosan was found to cause discomfort in human patients and required analgesic treatment (unpublished results). Thus, the aim of our study was to establish whether the cationic poly (amino acid) poly-L-lysine (PLL), which can be dispersed at physiological pH value, could be used as a desquamation inducer of the urothelium as an alternative to chitosan. We aimed to establish how the urothelial barrier function and structure are influenced by the polymer’s concentration, molecular weight, and treatment time. To be used as a desquamation inducer, poly-L-lysine should mainly exfoliate the superficial urothelial cells, and rapid restoration of urothelial function and structure should occur after the treatment. The function of the urothelium was evaluated ex vivo by measuring transepithelial electrical resistance using side-by-side diffusion cells (Ussing chambers), and urothelial structure was analyzed after ex vivo experiments and after in vivo exposure to poly-L-lysine by scanning electron microscopy.

## 2. Material and Methods

### 2.1. Materials

Poly-L-lysine hydrobromide (PLL) with molecular weights 30–70 kDa and 70–150 kDa were obtained from Sigma–Aldrich Chemie (Steinheim, Germany). Dispersions of PLL at 0.001% and 0.01% (*w*/*v*) were prepared in a phosphate buffer (PB), which consisted of 0.67 g of Na_2_HPO_4_, 0.19 g of KH_2_PO_4_, and 8.00 g of NaCl in 1 l of deionized water. The pH of the dispersions was adjusted to 6.5.

### 2.2. Animals for Ex Vivo Experiments

Nine young adult (8 to 9 weeks old) female Wistar rats (Harlan, Italy) were used for ex vivo experiments. The rats were housed at the Medical Experimental Center at the Faculty of Medicine (Ljubljana, Slovenia) in open-bar cages (two to five per cage) with bedding (Lignocel 3/4, Germany) and under controlled conditions of temperature (22 ± 1 °C) and humidity (55 ± 10%), and a 12 h/12 h light–dark cycle (light from 7 am to 7 pm) with unlimited access to food (standard rodent diet; Mucerola, Italy) and tap water. All procedures involving rats were approved by the National Ethical Committee and the Administration of the Republic of Slovenia for Food Safety, Veterinary and Plant Protection (permit number U34401-4/2013/3). Animal care and treatment were in accordance with Slovenian and international legislation and policy (Directive 2010/63/EU) on the protection of animals used for research purposes.

### 2.3. Ex Vivo Experiments

After the rats were sacrificed by CO_2_ inhalation, their urinary bladders were isolated and immediately placed in Dulbecco’s modified Eagle’s cell culture medium (DMEM; Sigma–Aldrich Chemie, Steinheim, Germany). The corpus of the urinary bladder was halved and the tissue was first placed onto an insert with a circular aperture (3 mm diameter, 0.07 cm² exposure area). Of the two rodent species only rat urinary bladders were used for transepithelial electrical resistance (TEER) measurement, because bladder halves of these animals entirely covered the aperture in the insert of the diffusion chamber and by this enabled the TEER measurements. Mouse bladders were found to be too small and the TEER measurements were not possible due to leakage at the edge of the aperture in the insert. No pharmacological agents were used to relax the bladder smooth muscles. The insert was then placed between EasyMount^®^ half-chambers of an Ussing chamber (Physiologic Instruments, San Diego, CA, USA). In diffusion chambers, the temperature was maintained at 37 °C and the incubation solutions were constantly oxygenated and stirred by gas (95% O_2_, 5% CO_2_).

**Figure 1:**
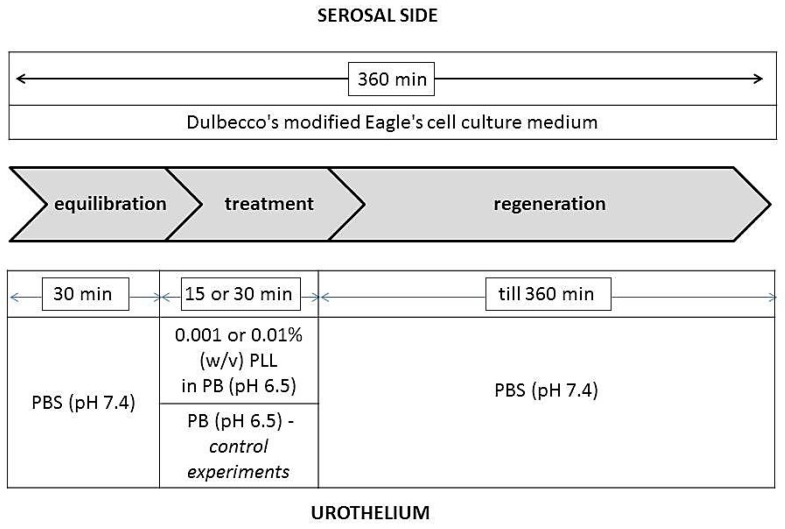
Schematic outline of the ex vivo experiments. Poly-L-lysine (PLL) with molecular weight 30–70 kDa or 70–150 kDa was used. PB = phosphate buffer.

During the entire duration of the experiments, the serosal side of the tissue was exposed to DMEM, and the urothelium was exposed to various media as shown in Figure 1. During the treatment period, the urothelium was exposed to a 0.001% or 0.01% (*w*/*v*) dispersion of PLL with pH 6.5 and a molecular weight of 30 to 70 kDa or 70 to 150 kDa. In the control experiments, the urothelium was exposed to PB with pH 6.5 because PLL dispersions were prepared in this buffer. The duration of the treatment period was 15 or 30 min. The experiments were performed in three or four replicates. 

In the ex vivo experiments with the most promising results, the tissue structure was also analyzed. The morphological evaluation of urothelial desquamation was performed after the treatment period and at the end of the regeneration period. At these particular time points, the tissue was removed from the insert of the diffusion chamber and prepared for observation by scanning electron microscopy.

#### Measuring of Transepithelial Electrical Resistance (TEER)

During the ex vivo experiment, the potential difference (*PD*) and passing current (*I*) were measured by a multi-channel voltage-current clamp (model VCC MC6, Physiologic Instruments, San Diego, CA, USA). Two pairs of Ag/AgCl electrodes were connected to the diffusion chambers via 3 M KCl/3.5% agar bridges. The experiments were performed under open circuit conditions with the current set to zero. For each measurement, a *PD* value was clamped to 20 mV and the necessary current was recorded. Electrical resistance was calculated according to Ohm’s law (*R* = (20 mV – *PD*)/*I*). To obtain TEER, the fluid resistance measured before mounting the tissue in the diffusion chamber was first subtracted and the value obtained was then multiplied by the exposure area (0.07 cm²).

Electrophysiological parameters were recorded during the equilibration period at 10 min intervals, in the treatment period at 5 min intervals, and during the regeneration period initially at 10 min intervals (up to 120 min) and then at 20 min intervals. Because the TEER values determined already varied considerably during the equilibration period, all measurements were presented as a percentage of the TEER value at the end of the equilibration period for each tissue separately. This ensured better comparability and repeatability of data. A two-tailed unpaired Student’s *t*-test (α = 0.05) was applied to evaluate the differences in TEER values between different experimental conditions at particular time points.

### 2.4. Animals for In Vivo Experiments

For the in vivo experiments, we used ten adult (10 to 12 weeks old) female C57BL/6JOLaHsd mice (Harlan, Italy), six mice for experiments with 0.01% PLL of both molecular weights and four mice for experiments with 0.001% PLL of both molecular weights. The mice were housed at the Medical Experimental Center at the Faculty of Medicine (Ljubljana, Slovenia) in open-bar cages (two to five per cage) with bedding (Lignocel 3/4, Germany) and enrichment material (paper towel) and under controlled conditions of temperature (22 ± 1 °C) and humidity (55 ± 10%), and a 12 h/12 h light–dark cycle (light from 7 am to 7 pm) with unlimited access to food (standard rodent diet; Mucerola, Italy) and tap water. All procedures involving mice were approved by the National Ethical Committee and the Administration of the Republic of Slovenia for Food Safety, Veterinary and Plant Protection (permit number U34401-4/2016/8). Animal care and treatment were in accordance with Slovenian and international legislation and policy (Directive 2010/63/EU) on the protection of animals used for research purposes.

### 2.5. In Vivo Experiments

**Figure 2:**
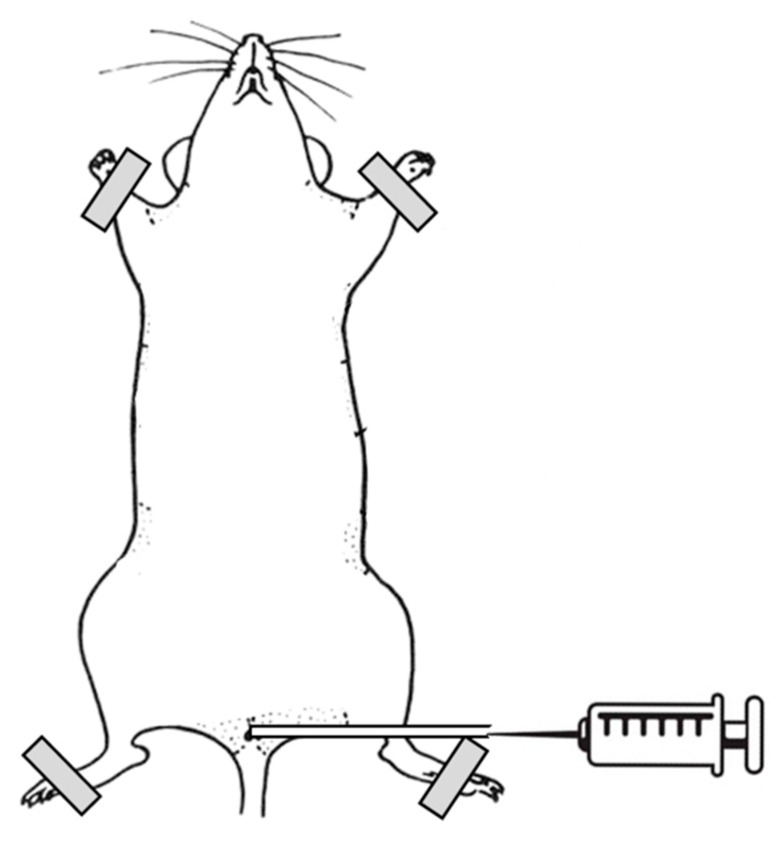
Schematic design of in vivo experiments.

The mice were anesthetized intraperitoneally with ketamine HCl (100 mg/kg) and xylazine (10 mg/kg), and placed in the dorsal position. A polyethylene catheter with a 0.28 mm inner diameter (Intramedic, Becton Dickinson, USA) was inserted into the bladder through the urethra and sheathed over a 29 G needle (Micro-Fine Plus^TM^, Becton Dickinson, USA) connected to a 1 mL injection syringe. The bladder of each animal was emptied with mild manual pressure on the abdomen. Then, 80 µl of PLL dispersion (30–70 kDa or 70–150 kDa, 0.001% or 0.01%) were instilled intravesically for 15 min (Figure 2). All infusions of PLL were performed gradually and at a slow rate to avoid injury or vesicoureteral reflux. After the elapsed time, the catheter and injection syringe were removed from the bladder and the mouse was sacrificed with CO_2_. The abdomen was opened, and the fully filled and distended urinary bladder was ligated with surgical thread. The bladder was then excised from the animal, cut longitudinally into halves, and further prepared for scanning electron microscopy.

### 2.6. Scanning Electron Microscopy

The tissue from the ex vivo and in vivo experiments was fixed in a mixture of 2% paraformaldehyde (Merck KgaA, Germany) and 2% glutaraldehyde (Serva, Germany) in 0.1 M cacodylate buffer (pH 7.4; Serva, Germany) for 3 to 4 h at 4 °C. The tissue samples were then rinsed in 0.1 M cacodylate buffer and post-fixed in 1% osmium tetroxide (Serva, Germany) in the same buffer for 1 h at 4 °C. Specimens were critical-point dried, attached to aluminum holders with silver paste (Silver DAG1415, Plano GmbH, Germany), sputter-coated with gold, and examined in a Tescan Vega3 scanning electron microscope at 30 kV (Brno, Czech Republic).

## 3. Results

### 3.1. Results of Ex Vivo Experiments

#### 3.1.1. Evaluation of Urothelial Function

**Figure 3:**
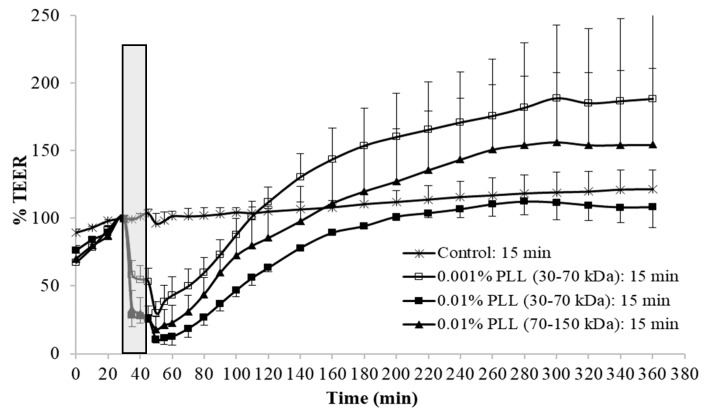
Average values and standard deviations of relative TEER of the urothelium exposed for 15 min to 0.001% or 0.01% poly-L-lysine (PLL) with a molecular weight of 30–70 kDa or 70–150 kDa. At each experimental condition, measurements were performed on urinary bladders of three to four different animals. Exposure time to PLL is marked gray.

**Figure 4:**
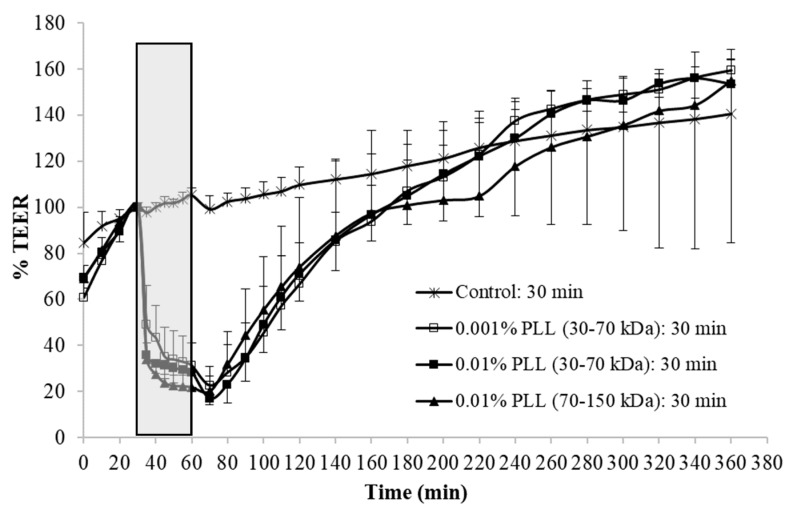
Average values and standard deviations of relative TEER of the urothelium exposed for 30 min to 0.001% or 0.01% poly-L-lysine (PLL) with a molecular weight of 30–70 kDa or 70–150 kDa. At each experimental condition measurements were performed on urinary bladders of three different animals. Exposure time interval to PLL is marked gray.

Gradual increase of TEER values was constantly observed in the equilibration period of the measurements, which can be explained by the tissue recovery after surgical stress induced by the excision of urinary bladder from the animal. The PLL was dispersed in PBS with a pH of 6.5, and, when the urothelium was exposed in the treatment period to this buffer only (control experiments), no considerable decrease in TEER was detected. During the regeneration period, the urothelium was incubated in PBS with a pH of 7.4 for 5 h, and in the meantime the TEER of the control tissue slowly but constantly increased (Figure 3 and Figure 4).

In the experiments with PLL, the molecular weight and concentration of polymer varied as well as the treatment time. The exposure of the urothelium to PLL always resulted in a significant decrease in TEER values compared to the control tissue, and during the regeneration TEER values gradually increased to the control values, but with a different rate (Figure 3 and Figure 4).

At the end of the treatment period, 15 min exposure to 0.001% PLL decreased TEER significantly less than the treatment with the higher concentration of PLL of the same molecular weight (*t*-test, *p* < 0.05). Consequently, the regeneration of TEER was also the fastest and the control values of TEER were already reached approximately 1 h after the end of the exposure to PLL. When the urothelium was exposed for 15 min to 0.01% PLL, the polymers with lower and higher molecular weight had a similar influence on the permeability of the urothelium during the treatment period (*t*-test, *p* > 0.05), but TEER restoration was faster after exposure to PLL with a higher molecular weight. It is interesting that even when PLL decreased TEER to approximately 10% of the baseline value, TEER returned to the control values by the end of the regeneration period (Figure 3).

At a longer treatment period (30 min), there were no significant differences in TEER at the end of the treatment period (*t*-test, *p* > 0.05) regardless of the PLL molecular weight and concentration tested. The TEER recovery was also comparable under all three treatment conditions (Figure 4).

#### 3.1.2. Morphological Evaluation of Urothelial Desquamation

Based on the results of TEER measurements, morphological evaluation was performed for the tissue exposed in ex vivo conditions for 15 min to 0.001% PLL or 0.01% PLL with a molecular weight 30 to 70 kDa. The urothelial tissue was removed from the diffusion chamber and prepared for morphological observations at the ends of treatment and regeneration periods.

**Figure 5:**
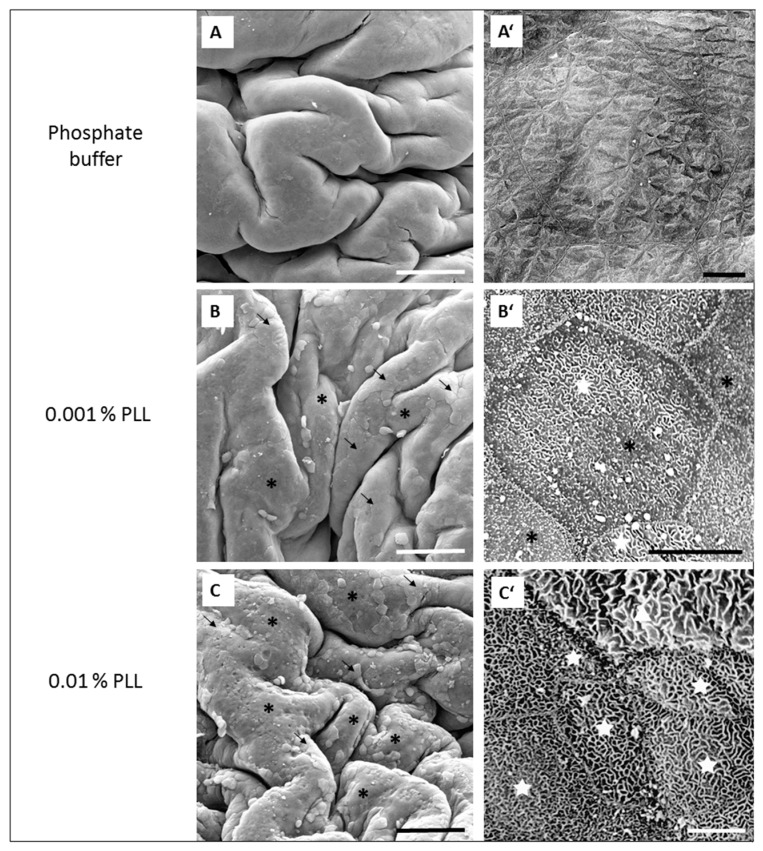
Representative SEM micrographs of the urothelium at the end of the 15 min treatment period with phosphate buffer (**A** and **A’**), with 0.001% PLL (30–70 kDa) (**B** and **B’**), or with 0.01% PLL (30–70 kDa) (**C** and **C’**) in ex vivo experiments. Images (**A’**, **B’** and **C’**) show key features of the urothelium at higher magnification. Image **A** shows intact urothelium and image **A’** shows polygonal terminally differentiated superficial cells (umbrella cells) with typically scalloped apical plasma membrane composing luminal surface of intact urothelium. In images **B** and **C**, the majority of the urothelium is desquamated (black asterisks) and in only some areas are umbrella cells still present (black arrows). In images **B’** and **C’**, desquamated areas of the urothelium are presented under higher magnification, where cells with microvilli (black asterisks in **B’**) and ropy ridges of apical membrane (white stars in **B’** and **C’**) are exposed on urothelial surface due to desquamation of umbrella cells. Only individual umbrella cells still remain on the urothelial surface after such treatment (white triangle in **C’**). Scale bars: 200 µm (**A**,**B**,**C**), 20 µm (**A’**), and 10 µm (**B’**,**C’**).

After 15 min exposure to PBS with pH 6.5 (control experiments), the luminal surface of the urothelium remained unchanged (Figure 5A,A’). At the end of 15 min of treatment with 0.001% PLL, cell desquamation was present only to a small extent and only superficial cells were found to peel off (Figure 5B,B’). In desquamated areas, smaller and less differentiated former intermediate cells with microvilli or ropy ridges of apical plasma membrane were exposed on the urothelial surface. Cleaved tight junctions between superficial cells were observed in early steps of superficial cell desquamation. At the end of the treatment period with 0.01% PLL, much more extensive cell desquamation was found in comparison to the treatment with 0.001% PLL because the majority of the urothelium was peeled off. Cell desquamation was restricted to superficial cells only, and newly exposed intermediate cells predominated on the urothelial surface (Figure 5C,C’).

**Figure 6:**
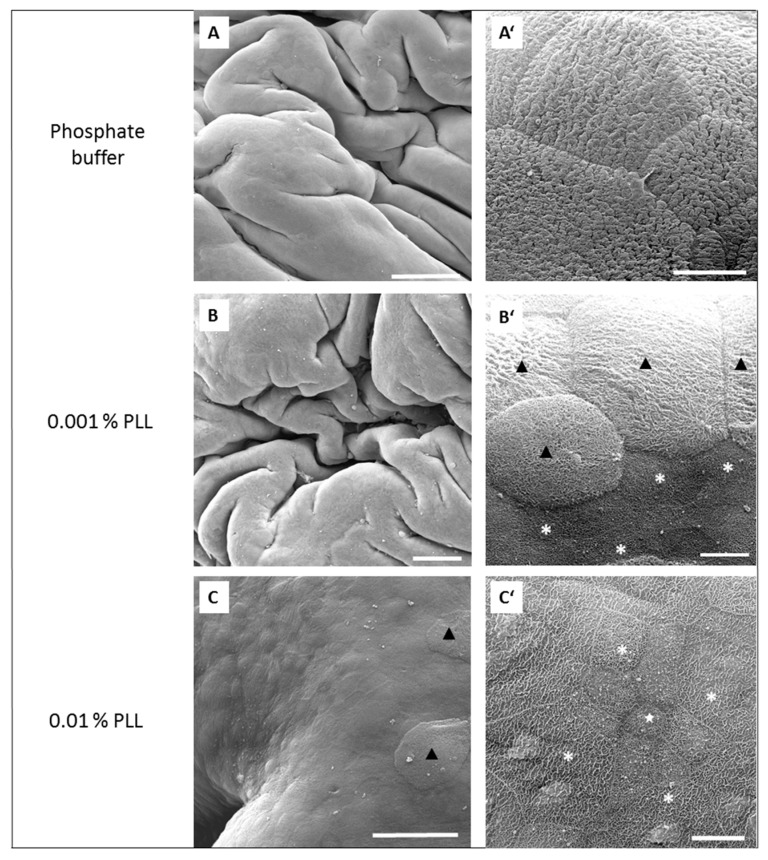
Representative SEM micrographs of the urothelium at the end of the regeneration period after the urothelium was exposed to phosphate buffer (**A** and **A’**), 0.001% PLL (30–70 kDa) (**B** and **B’**) or to 0.01% PLL (30–70 kDa) (**C** and **C’**) in ex vivo experiments. Images **A’**, **B’** and **C’** show key features of the urothelium at higher magnification. Luminal surface of intact urothelium (**A**) consists of large, terminally differentiated superficial cells (umbrella cells) with specifically scalloped apical plasma membrane (**A’**). In images **B**, **B’**, **C**, and **C’**, the whole urothelial superficial layer was renewed after desquamation and composed of rare small cells with microvilli (white star in **C’**) and predominant cells with ropy ridges of apical plasma membrane (white asterisks in **B’** and **C’**) between undesquamated umbrella cells (black triangles in **C** and **B’**). Scale bars: 200 µm (**A**,**B**), 50 µm (**C**), 20 µm (**A’**), and 10 µm (**B’**,**C’**).

At the endpoint of the experiment (at 360 min), which was also at the end of the regeneration period, the urothelium from the control experiments was intact (Figure 6A,A’). However, also after the exposure of the urothelium to 0.001% PLL, no desquamation of the urothelium was observed at the end of the regeneration period. The superficial cell layer was composed of old umbrella cells, which had not desquamated, and new superficial cells, which replaced previously desquamated umbrella cells due to the PLL treatment. These newly exposed superficial cells were at a lower stage of cell differentiation than the old ones, and most of them had ropy ridges on their apical surface (Figure 6B,B’). Also, after exposure to 0.01% PLL, the urothelial structure was renewed at the end of the regeneration period, with no cell desquamation. Newly exposed intermediate cells were at a higher stage of cell differentiation than at the end of the treatment period. All of them had ropy ridges on their apical surface (Figure 6C,C’).

### 3.2. Results of In Vivo Experiments

#### Morphological Evaluation of Urothelial Desquamation

**Figure 7:**
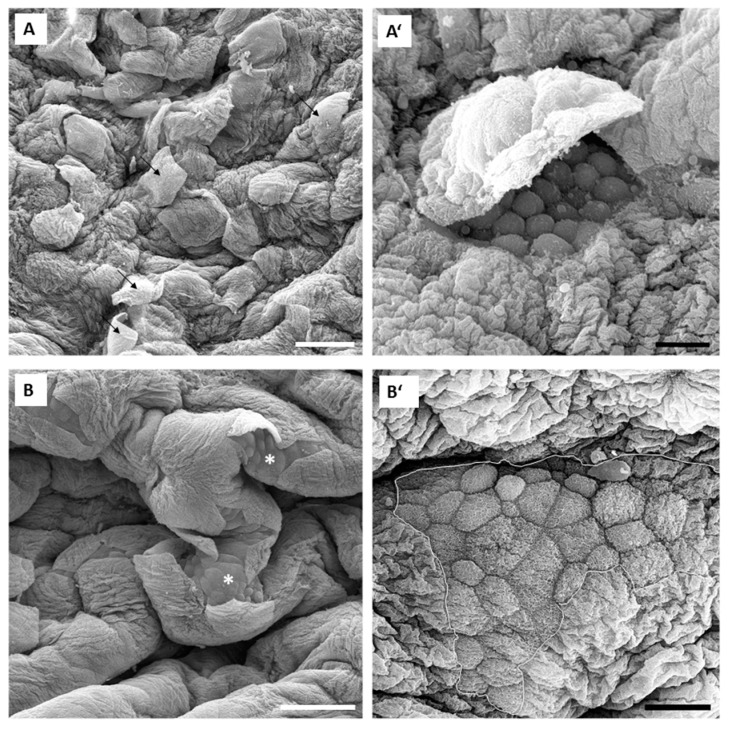
Representative SEM micrographs of the urothelium in in vivo experiments after 15 min exposure to 0.001% PLL with molecular weights 30–70 kDa (**A** and **A’**) and 70–150 kDa (**B** and **B’**). Images **A’** and **B’** show key features of the urothelium at higher magnifications. In image **A**, only individual umbrella cells desquamate (black arrows), while in image **B** small groups of umbrella cells desquamate or have already been peeled off causing occasional small desquamated areas of the urothelium (white asterisks). Images **A’** and **B’** show desquamated areas of the urothelium (encircled with a white line in **B’**). Exposed former intermediate cells are smaller than umbrella cells and without scalloped apical plasma membrane revealing lower differentiation stage. Scale bars: 100 µm (**A**,**B**), 20 µm (**A**’,**B**’).

Morphological examination of the urothelium, which was exposed for 15 min to 0.001% PLL with a molecular weight of 30 to 70 kDa, revealed desquamation of only individual umbrella cells (Figure 7A,A’). In the urothelium exposed to 0.001% PLL with molecular weight 70–150 kDa, cell desquamation was found in only a few small areas of the urothelium, whereas the majority of urothelial surface was intact. Only individual umbrella cells or small groups of them underwent desquamation (Figure 7B,B’). 

**Figure 8:**
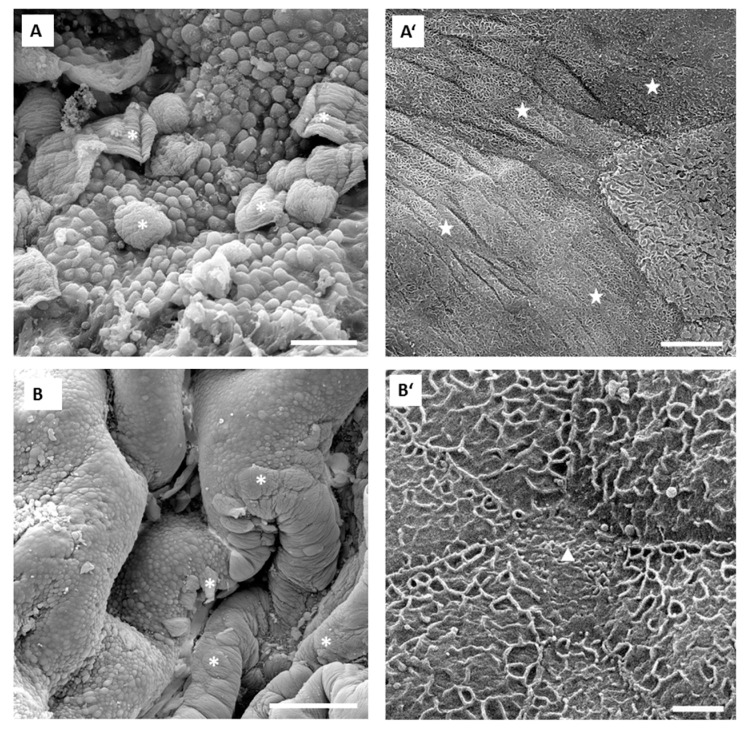
Representative SEM micrographs of the urothelium in in vivo experiments after 15 min exposure to 0.01% PLL with molecular weight 30–70 kDa (**A** and **A’**) and 70–150 kDa (**B** and **B’**). Images **A’** and **B’** show key features of the urothelium at higher magnifications. In images **A** and **B** are seen large and predominant areas of the urothelium, where umbrella cells desquamated and, thus, intermediate cells are exposed on the surface. Some still attached umbrella cells, which did not undergo desquamation, are present on urothelial surface (white asterisks). In desquamated urothelial areas in images **A’** and **B’**, exposed intermediate cells as new superficial cells are small and with microvilli (white triangle in **B’**) or ropy ridges of apical plasma membrane (white stars in **A’**). Scale bars: 50 µm (**A**), 200 µm (**B**), 20 µm (**A’**), and 2 µm (**B’**).

Morphological analysis of the urothelium, which was exposed for 15 min to 0.01% PLL with a molecular weight of 30 to 70 kDa, showed intense desquamation. In extensive desquamated areas of the urothelium, desquamation took place in the superficial cell layer only. Therefore, small and partially differentiated former intermediate cells with microvilli or ropy ridges of apical plasma membrane were exposed on the urothelial surface (Figure 8A,A’). In the urothelium exposed to 0.01% PLL with a molecular weight of 70 to 150 kDa, intense cell desquamation was found and the majority of urothelial luminal surface was peeled off. Due to the desquamation of exclusively superficial cells, urothelial surface was composed of exposed former intermediate cells. These cells were smaller and with microvilli or ropy ridges of apical plasma membrane revealing a lower cell differentiation stage in comparison to desquamated superficial cells [17]. No desquamation into deeper urothelial cell layers was detected (Figure 8B,B’).

## 4. Discussion

Cell desquamation is part of the physiological renewal in some tissues (e.g., the epidermis), but it can be enhanced by various inducers or types of stress conditions [18,19]. The urothelium is a very stable epithelium, but it quickly responds to various pathological stimuli with intense cell desquamation [4,5,20]. However, the desquamation should be limited to the upper urothelial cell layers, without provoked bleeding, and has to be reversible. Namely, rapid regeneration of urothelial function and structure is essential to restore the blood–urine barrier. Our previous ex vivo and in vivo studies showed that chitosan induces desquamation of the urothelial cells, with rapid regeneration of the urothelium after the removal of the polymer [4,5], and that its effects on urothelial structure and function are time- and concentration-dependent [12]. The aim of this study was to establish whether another cationic polymer, PLL, affects the urothelium similar to chitosan and could be used as an alternative desquamation inducer. In contrast to chitosan, which has to be dispersed in a buffer with acidic pH value, PLL can be dispersed in a buffer at close to neutral pH value. This could be important to reduce discomfort of human patients during potential intravesical application by clinicians. 

A PLL with a molecular weight from 30 kDa to 300 kDa was used in the studies where epithelial permeability was examined [16]. It has also been used in urinary bladder pretreatment in orthotopic bladder tumor model to enhance the electrostatic interactions between tumor cells and the urothelium to increase the tumor implantation rate [21,22]. However, the results of our previous study proved that PLL (molecular weight 70–150 kDa, 0.01% concentration) caused desquamation of urothelial cells and, therefore, enabled tumor cells to attach and implant, which is in accordance with the findings of the present study [23].

In ex vivo experiments performed with Ussing chambers, we aimed to establish how the concentration and molecular weight of PLL as well as treatment time influence the permeability of the urothelium. During these experiments, TEER was determined, which is a sensitive measure of the ion permeability of the urothelium [2]. Our previous studies already proved that in ex vivo experiments with Ussing chambers, the urinary bladders isolated from animals maintain the barrier function and cellular ultrastructure for at least 6 h [5]. This was also confirmed in this study because at the end of the experiment (at 360 min) the TEER was higher than at the beginning of the measurement, and the luminal surface of the urinary bladders was intact, with no observable damage or cell death (Figure 5E). 

When the urothelium was exposed for 30 min to 0.01% PLL of different molecular weight (30–70 kDa or 70–150 kDa), there was a similar drop in TEER, followed by a comparable rate of TEER restoration (Figure 4). At a shorter exposure time (15 min), there was also no significant difference in drop of TEER during the exposure of the urothelium to 0.01% PLL of a different molecular weight. However, after treatment with PLL of a higher molecular weight, TEER restoration was slightly faster (Figure 3). 

At the end of the treatment period, drop in TEER was significantly smaller when the urothelium was exposed for 15 min to 0.001% PLL compared to either a higher concentration of PLL (0.01%) or longer exposure time (30 min; Figure 3 and Figure 4). Only the minority of the superficial cells were exfoliated at the end of 15 min exposure to 0.001% PLL (Figure 5B), and this treatment with PLL would probably not be enough to remove the reservoirs of *Escherichia coli* in the superficial urothelial cells.

Fifteen-minute exposure to 0.01% PLL or 30 min exposure to either concentration of PLL resulted in the same reduction of TEER at the end of the treatment period (approximately 10 to 30% of the baseline value). Morphologically, 15 min exposure of the urothelium to 0.01% PLL resulted in extensive umbrella cell desquamation. However, it is important that the desquamation was limited to the superficial cells only (Figure 5C,C’). With more extensive desquamation of urothelial cells, more time was needed during the regeneration period to attain the TEER values of the control tissue. After 15 min treatment with 0.01% PLL, TEER values reached the control values at approximately 2.5 to 3 h after the end of the treatment, and at the end of the regeneration period no cell desquamation was observed. Newly exposed intermediate cells were not finally differentiated, but they were already at a higher stage of cell differentiation compared to the end of the treatment period (Figure 6C,C’). This is in agreement with the fact that urothelial regeneration after induced injury is much faster than physiological renewal [4,5,24].

In the regeneration period the urothelium recovered from induced desquamation. The rate of recovery was different among tissue pieces, which is expected in biological samples. Moreover, TEER values of the control tissue also gradually increased throughout the experiment, while the relative TEER values were normalized to the TEER value at the end of the equilibration period. Both facts increase the standard deviations of the measured values at the later time points. In some cases, TEER values at the end of ex vivo experiments were even higher compared to the values at the end of the equilibration period. However, as shown in our previous study, this was not a consequence of hyperplasia [5].

Therefore, from the ex vivo experiments we concluded that 15 min treatment with 0.01% PLL yields promising results for potential use of PLL as a desquamation inducer of the urothelium. The influence of PLL on urothelial structure and function is mostly influenced by PLL concentration, whereas the treatment time and the molecular weight of the polymer had a minor effect. Comparing the influence of chitosan on the permeability of isolated pig urothelium, it was time and concentration dependent [12].

We evaluated urothelial desquamation and regeneration using TEER measurement as a quantitative method on the one hand, and analyzed the urothelial structure by scanning electron microscopy on the other hand. By these two methods we examined the level of functional and structural regeneration of this tissue after induced desquamation. Scanning electron microscopy has been proven in our previous studies [5,17] as the most appropriate method to detect changes in the differentiation stage of urothelial cells. Namely, differentiation levels of these cells are best reflected in the structure of apical plasma membrane. The structuration of the apical plasma membrane into microvilli (cells at low differentiation stage), ropy ridges (cells at high differentiation stage) or specifically scalloped surface (terminally differentiated cells) has been shown as a relevant marker of urothelial cell differentiation [4,5,17]. Our results obtained in in vivo experiments are in accordance with the results of ex vivo experiments performed with isolated urinary bladders. In in vivo conditions, 0.001% concentration of PLL was also too low for the desired effect. Namely, only individual umbrella cells were exfoliated when the urothelium was exposed to 0.001% PLL with molecular weights of either 30 to 70 kDa or 70 to 150 kDa (Figure 7). On the other hand, exposure to 0.01% PLL resulted in intense cell desquamation that was limited to the superficial cell layer (Figure 8), with no apoptotic or necrotic cells on the urothelial surface. 

In the in vivo experiments with 0.01% PLL, the molecular weight of the polymer influenced the desquamation effect because at a higher molecular weight the majority of the urothelium was exfoliated (Figure 8). The PLL with a higher molecular weight has a larger number of amino groups, which are positively charged at pH 6.5 because the pKa of PLL is approximately 10.5 [25]. Consequently, we expected better contact between such PLL with higher density of positively charge and negatively charged urothelial surfaces. Namely, the urothelium is covered with the glycosaminoglycan layer, mainly composed of heparin, dermatan, chondroitin sulphate, and hyaluronic acid [26]. On the other hand, there was no significant difference between 0.01% PLL of 30–70 kDa and 70–150 kDa on urothelial permeability (Figure 4), while in the experiments performed with the human intestinal Caco-2 cell line, PLL of a higher molecular weight (300 kDa) had a significantly greater effect on TEER and inulin passage compared to PLL with a molecular weight of 30 kDa [16]. The different effects of PLL on epithelial permeability could be explained by the different structures of both epithelia. The urothelium is a three-layered epithelium with complex architecture, whereas the Caco-2 cell line grows as a monolayer. The insignificant influence of PLL molecular weight on urothelium permeability might be also due to the relatively small difference between the molecular weights tested in our study. However, in the in vivo experiments with 0.01% PLL more intense cell desquamation was present with a molecular weight of 70–150 kDa compared to molecular weight of 30–70 kDa (Figure 7). Consequently, it would not be rational to use a PLL with an even higher molecular weight. 

Therefore, 15 min exposure of the urothelium to a 0.01% concentration of PLL results in uniform desquamation of the superficial cell layer, followed by rapid regeneration of the urothelium. In comparison to chitosan, the main advantage of PLL is that the desquamation is limited to only a superficial cell layer. Namely, in comparable ex vivo experiments, 15 min exposure of the urothelium to 0.005% chitosan resulted in intense desquamation of superficial cells, but intermediate cells were also removed to some extent [5]. In addition, in the experiments performed with Caco-2 cells, TEER recovery was much faster after PLL treatment compared to chitosan when the same concentration of polymers was used [16].

The mechanism by which PLL induces desquamation of urothelial cells is not known. No studies have yet been published evaluating the effect of this polymer on urothelial permeability and structure. In Caco-2 cells, PLL influenced tight junctions through morphological changes of the F-actin cytoskeleton and redistribution of the tight junction proteins ZO-1 and occludin [16].

It is also worth mentioning that the results of ex vivo experiments are in accordance with the results obtained by in vivo experiments despite the use of the tissue from two different types of animals. This confirms the fact that rat and mouse urothelium have very similar structures and similar responses to PLL. Additionally, we can expose that ex vivo experiments performed with Ussing chambers may replace in vivo experiments when the main focus of the research is the influence of a substance on urothelial permeability at different treatment conditions. In view of this, the concept of our study supports the basic principles of the 3Rs in the use of animals for research purposes. 

## 5. Conclusions

Based on our results of ex vivo and in vivo experiments, we can conclude that 15 min exposure of the urothelium to 0.01% PLL is effective and safe at the same time. The PLL induced intense cell desquamation limited only to the superficial cell layer, followed by rapid regeneration. The influence of PLL on urothelial structure and function was mostly influenced by PLL concentration, whereas the treatment time and molecular weight of the polymer had minor effects. Consequently, we can conclude that PLL is a better alternative to chitosan regarding its potential use as a desquamation inducer of the urothelial cells.
